# Effects of Three Different Exercise Strategies for Optimizing Aerobic Capacity and Skeletal Muscle Performance in Older Adults: A Pilot Study

**DOI:** 10.14283/jfa.2021.21

**Published:** 2021-05-14

**Authors:** Dallin Tavoian, D. W. Russ, T. D. Law, J. E. Simon, P. J. Chase, E. H. Guseman, B. C. Clark

**Affiliations:** 1 Ohio Musculoskeletal and Neurological Institute (OMNI), Ohio University, Athens, USA; 2 School of Physical Therapy and Rehabilitation Sciences, University of South Florida, Tampa, FL USA; 3 School of Applied Health Sciences and Wellness, Athens, USA; 4 Diabetes Institute, Bethesda, USA; 5 Department of Primary Care, Baton Rouge, USA; 6 Department of Biomedical Sciences, Dothan, USA; 7 Division of Geriatric Medicine at Ohio University, Athens, OH USA

**Keywords:** High-intensity interval training, exercise, aging, physical function, muscle

## Abstract

This Brief Report describes a pilot study of the effect of 12 weeks of stationary bicycle high-intensity interval training, stationary bicycle moderate-intensity continuous training, and resistance training on cardiorespiratory, muscular, and physical function measures in insufficiently-active older adults (N=14; 66.4±3.9 years; 3 male, 11 female). After baseline testing, participants were randomly assigned to one of the exercise groups. High-intensity interval training and moderate-intensity continuous training had small-to-large effect sizes on cardiorespiratory/endurance and physical function measures, but very small effect sizes on muscular measures. Resistance training had small-to-large effect sizes on cardiorespiratory, muscular, and physical function measures. This pilot study should be interpreted cautiously, but findings suggest that resistance exercise may be the most effective of the three studied exercise strategies for older adults as it can induce beneficial adaptations across multiple domains. These effect sizes can be used to determine optimal sample sizes for future investigations.

**Electronic Supplementary Material:**

Supplementary material is available for this article at 10.14283/jfa.2021.21 and is accessible for authorized users.

## Introduction

**D**espite well-documented muscular and cardiorespiratory health benefits that accompany regular exercise participation, most older adults are not engaging in exercise with the volume and/or intensity sufficient for maintaining physical function (1, 2). In fact, fewer than 13% of older adults meet the aerobic (150 minutes moderate intensity/week; e.g., walking, stationary bicycling) and muscle strengthening (2 days/week; e.g., weight lifting) guidelines concurrently, while only 31% meet one of the two (3). A more pragmatic approach that emphasizes a single exercise strategy with the greatest effect on overall health may be a reasonable solution to optimize outcomes and improve adherence (4).

High-intensity interval training (HIIT) is an exercise strategy consisting of short periods (10 seconds to 4 minutes) of vigorous exercise interspersed with low-intensity rest periods. It can improve cardiorespiratory fitness and lower cardiovascular disease risk equal to, or greater than, traditional aerobic training (5), and has also been shown to improve muscle strength in young adults (6). However, the potential for HIIT to induce muscular benefits in older adults has not been adequately explored. The aim of this study was to examine whether stationary bicycle HIIT was a more efficient standalone exercise strategy to improve cardiovascular and lower extremity muscular function than established muscle strengthening (resistance training; RT) or aerobic (moderate-intensity continuous training; MICT) programs in older adults.

## Methods

An in-depth protocol for this study has been published previously (7), and only essential information is provided in this section. It should be noted that a sample size of 24 (n=8/group) was initially planned for this pilot study. However, restrictions on human subjects research associated with the COVID-19 pandemic prevented attainment of the recruitment goal. Thus, we only present descriptive statistics and effect size estimates in this Brief Report.

### Participant characteristics

Twenty-two generally healthy but insufficiently active (i.e., not meeting either aerobic or muscle strengthening guidelines (7)) participants aged 60–75 years were recruited, enrolled, and randomized, with 14 (66.4 ± 3.9 years; 3 male, 11 female) completing the study. One was removed for starting a new blood pressure medication while on the study protocol, and seven others were interrupted prior to completion due to the COVID-19 pandemic and unable to resume the study. Written informed consent was obtained from each participant in accordance with the Declaration of Helsinki. Ethical Approval for this study has been obtained from the Ohio University Institutional Review Board. Baseline characteristics are shown in Table 1.

**Table 1:** Baseline and post-intervention characteristics

	**HIIT**			**MICT**			**RT**		
	**Pre**	**Post**	**ES**	**Pre**	**Post**	**ES**	**Pre**	**Post**	**ES**
Descriptive Characteristics									
Age (years)	66.0 ± 3.3	-	-	65.3 ± 4.5	-	-	67.8 ± 4.5	-	-
N (% Female)	5 (80)	-	-	4 (75)	-	-	5 (80)	-	-
Body Mass (kg)	76.1 ± 19.7	75.9 ± 18.4	-	86.7 ± 30.6	88.0 ± 31.8	-	72.5 ± 15.7	71.2 ± 16.3	-
BMI (kg/m^2^)	28.3 ± 5.5	28.2 ± 5.0	-	30.4 ± 5.1	30.8 ± 5.4	-	27.6 ± 4.2	27.1 ± 4.0	-
Primary Outcomes									
Isokinetic Strength (N-m)	99.4 ± 23.0	99.5 ± 24.1	-0.01	105.9 ± 58.2	113.9 ± 64.4	0.11	94.5 ± 6.5	106.2 ± 25.0	0.56
Absolute VO2max (L/min)	1.46 ± 0.35	1.61 ± 0.27	0.44	1.91 ± 0.68	2.04 ± 0.68	0.16	1.41 ± 0.28	1.55 ± 0.33	0.41
Relative VO2max (mL/kg/min)	19.4 ± 1.6	21.7 ± 3.2	-	22.3 ± 3.2	24.0 ± 5.9	-	19.7 ± 2.7	22.2 ± 4.1	-
Muscle Volume (cm3)	411.4 ± 82.4	429.2 ± 86.3	0.19	432.3 ± 194.5	478.9 ± 192.3	0.21	425.9 ± 89.0	456.9 ± 107.0	0.28
Secondary Outcomes									
Isometric Strength (N-m)	129.8 ± 45.7	122.8 ± 32.6	-0.17	129.4 ± 58.4	127.0 ± 51.5	-0.04	117.5 ± 23.0	148.7 ± 32.4	0.99
Fatigue Resistance (% of maximal)	48.0 ± 9.7	57.6 ± 5.0	1.13	43.3 ± 8.0	54.0 ± 12.2	0.90	50.0 ± 10.6	55.6 ± 15.0	0.39
Fat Mass (kg)	29.6 ± 8.7	29.4 ± 8.3	0.02	31.3 ± 13.3	31.1 ± 13.6	0.02	25.9 ± 8.0	24.7 ± 7.1	0.14
Physical Function Outcomes									
6MW (m)	568.0 ± 34.2	611.2 ± 38.2	1.08	587.3 ± 56.1	600.5 ± 60.1	0.20	557.2 ± 60.3	585.0 ± 67.2	0.39
4SST (s)	6.41 ± 0.71	6.43 ± 0.42	-0.02	6.34 ± 0.79	5.60 ± 1.15	0.65	7.34 ± 2.16	6.60 ± 2.00	0.32
Grip Strength (kg)	26.2 ± 3.7	28.2 ± 3.4	0.51	31.9 ± 5.7	33.3 ± 8.7	0.17	26.2 ± 8.9	26.5 ± 10.3	0.12
Chair Rise (s)	8.80 ± 1.72	7.63 ± 2.82	0.50	9.39 ± 2.15	7.29 ± 1.20	1.21	9.62 ± 1.90	7.41 ± 2.24	1.07

Data are means ± SD. 4SST, four-square step test; 6MW, six-minute walk; BMI, body mass index; ES, effect size; HIIT, high-intensity interval training; MICT, moderate-intensity continuous training; RT, resistance training; VO2max, maximal oxygen consumption. Effect sizes are classified as very small (0.01–0.19), small (0.20–0.49), moderate (0.5–0.79), large (0.8–1.19), and very large (>1.20)

### Study Design

This study had a screening/baseline assessment period of three sessions, randomization into one of the three exercise groups, a 12-week exercise training period, and a post-intervention assessment period of two sessions (7). All exercises were performed on site three days per week and supervised by an exercise professional. Below we provide a brief description of the experimental procedures and training programs. We refer the reader to the Supplement as well as our previously published detailed protocol (7) for additional information.

### Procedures

#### Primary Outcomes

- Isokinetic Strength: Obtained at 60°/second from the non-dominant knee extensors.
- Maximal oxygen consumption (VO2max): Obtained during a graded cycle ergometry exercise test.
- Quadriceps muscle volume: Assessed from magnetic resonance imaging scans of the non-dominant leg.

#### Secondary Outcomes

- Isometric Strength: Obtained from the non-dominant knee extensors at 90° of knee flexion.
- Fatigue Resistance: Assessed through a series of 120 isokinetic leg extensions at 120°/second.
- Total Body Fat Mass: Obtained via whole-body dual-energy X-ray absorptiometry scans.

#### Physical Function Outcomes

- Six-Minute Walk (6MW): Completed on a 30-meter course.
- Four-Square Step Test (4SST): Performed in a four-foot by four-foot square split into quadrants.
- Grip Strength: Obtained with a Jamar hydraulic grip strength dynamometer at position II.
- Five-Time Chair Rise: Performed on a chair with the seat 18 inches from the ground.

### Exercise Intervention

Each participant performed their prescribed exercise 3x/week for 12 weeks. Adherence was defined as an attendance rate ≥80% (i.e., attended 29 of 36 exercise sessions), which all participants achieved. Participants in the HIIT group performed all exercises on a stationary bicycle (Peloton Interactive, Inc. New York City, NY, USA). The duration of the HIIT sessions were half the duration of the MICT sessions. Participants in the MICT group used the same stationary bicycle setup as in the HIIT group. Participants in the RT group performed all exercises using free weights, machines, or body weight.

### Statistical analysis

The planned analysis for this study was a one-way ANOVA to compare group means. However, because we could not complete the study due to COVID-19 our sample size is not adequately powered for this type of analysis. Therefore, descriptive statistics, percent change from baseline (primary and secondary outcomes), absolute change from baseline (physical function outcomes), and corrected Hedge’s g effect sizes for small samples are reported. Effect sizes were classified as very small (0.01–0.19), small (0.20–0.49), moderate (0.5–0.79), large (0.8–1.19), and very large (>1.20) (8). 95% confidence intervals for descriptive statistics can be found in the Supplemental Table S1.

## Results

**Figure 1:**
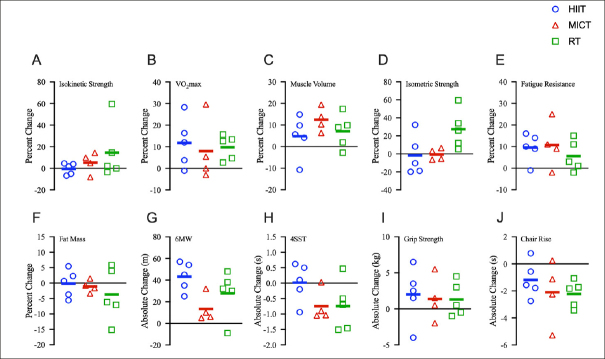
Changes in primary (A–C), secondary (D–F) and physical function outcomes (G–I) after 12 weeks of HIIT, MICT, or RT Open symbols are values for individual subjects and solid bars indicate group means. A) knee extensor isokinetic strength; B) absolute VO2max; C) muscle volume; D) knee extensor isometric strength; E) knee extensor fatigue resistance; F) total body fat mass; G) six-minute walk (6MW) distance; H) four-square step test (4SST) time; I) non-dominant hand grip strength; J) five-time chair rise time.

High-intensity interval training had very small effects on muscular strength and mass (ES=-0.17 to 0.19), small-to-large effects on cardiorespiratory/endurance measures (ES=0.44 to 1.13), and moderate-to-large effects on most physical function measures (ES=0.50 to 1.08). MICT had very small-to-small effects on muscular strength and mass (ES=-0.04 to 0.21), very small-to-large effects on cardiorespiratory/endurance measures (ES=0.16 to 0.90), and very small-to-very large effects on physical function (ES=0.17 to 1.21). RT had small-to-large effects on muscular strength and mass (ES=0.28 to 0.99), small effects on cardiorespiratory/endurance measures (ES=0.39 to 0.41), and very small-to-large effects on physical function (ES=0.12 to 1.07). All results can be found in Table 1 and Figure 1. See Supplement for detailed adverse event and adherence outcomes.

## Discussion

The purpose of this study was to compare the effect of stationary bicycle HIIT on cardiorespiratory/endurance and muscular strength and size measures, as well as physical function adaptations, to MICT or RT in generally healthy but insufficiently active older adults. Though terminated early due to COVID-19 restrictions, the diverse data that were collected allowed us to calculate effect sizes to power future investigations. First, HIIT had a greater effect on VO2max than MICT (ES=0.44 and 0.16, respectively), and a similar large effect on fatigue resistance (ES=1.13 and 0.90, respectively). MICT has long been promoted as an essential element in healthy aging (9), and it is becoming more and more clear that HIIT is also a safe aerobic exercise regimen that is highly effective at improving cardiac, respiratory, and metabolic function in an older adult population (10). A somewhat unexpected finding of this study, however, was the effect of RT on VO2max. The benefits of aerobic and resistance training have historically been considered independent of each other, and as such there has been relatively little attention given to the effects of RT on cardiorespiratory variables (4).

Stationary bicycling is an ideal form of aerobic exercise for older adults due to its effectiveness at inducing cardiorespiratory adaptations and the relative low risk of injury (11), and has also been shown to elicit strength improvements in older adults when used for MICT (12) or HIIT (13). We expected a similar response to our cycling protocols, however, our low-volume bicycle HIIT protocol had a very small effect on muscular strength and size at the group level. There was a diverse response to HIIT at the individual level—some participants showed substantial increases while others demonstrated substantial declines in muscle strength and size (Figure 1). It is unclear why our cycling protocols did not consistently result in improved strength, as has been reported previously (12, 13), although there are several methodological factors that may affect muscular adaptations (e.g., resistance, cadence).

Due to the relatively recent interest in HIIT for older adults there are few studies reporting effects on physical function measures, though those that do appear to indicate beneficial effects (13–15). This proof-of-concept pilot study demonstrates that HIIT had a large effect on 6MW distance and a moderate effect on grip strength and chair rise time, indicating that HIIT can improve physical functional capacity in older adults without overt physical function limitations. This may translate into substantial improvements in physical function capacity in mobility-limited older adults, and future work should investigate this possibility. In this study we chose a pragmatic approach wherein our participants followed national exercise guidelines; however, we should note that nuanced differences in training paradigms (e.g., different intensities or controlling for total volume, duration, or caloric expenditure) could have yielded different results.

## Conclusion

HIIT is a time-efficient exercise strategy that has the potential to produce both cardiorespiratory and muscular improvements, but few groups have investigated this potential. Our low-volume HIIT protocol did not consistently induce muscular adaptations but did elicit effects on cardiorespiratory/endurance and physical function measures comparable to MICT with half of the time commitment. Additionally, RT had small-to-moderate effects on cardiovascular/endurance measures along with the expected larger effects on strength. Future work should include strength and physical function measures to better characterize the adaptations to HIIT in order to determine if it is an effective and efficient exercise strategy for healthy and mobility-limited older adults.

## Electronic supplementary material

Supplement - Effects of Three Different Exercise Strategies for Optimizing Aerobic Capacity and Skeletal Muscle Performance in Older Adults: A Pilot Study

## Abbreviations

4SST: four-square step test
6MW: six-minute walk
ES: effect size
HIIT: high-intensity interval training
KE: knee extensor
MICT: moderate-intensity continuous training
RT: resistance training
VO2max: maximal oxygen consumption
