# Supplementary material for: Effects of Three Different Exercise Strategies for Optimizing Aerobic Capacity and Skeletal Muscle Performance in Older Adults: A Pilot Study
Source: J Frailty Aging. 2021 May 14;10(4):357–60. doi: 10.14283/jfa.2021.21 (PMC8114968; doi:10.14283/jfa.2021.21)
Supplement: Supplementary file 1 — Supplement - Effects of Three Different Exercise Strategies for Optimizing Aerobic Capacity and Skeletal Muscle Performance in Older Adults: A Pilot Study [file 42415_2021_109_MOESM1_ESM.docx]

Supplement - Effects of Three Different Exercise Strategies for Optimizing Aerobic Capacity and Skeletal Muscle Performance in Older Adults: A Pilot Study

Dallin Tavoian, David W. Russ, Timothy D. Law, Janet E. Simon, Paul J. Chase, Emily Hill Guseman, Brian C. Clark

**Outcome Measures**

See Tavoian et al. [1] for further descriptions of protocols.

*Knee extensor isokinetic strength*: Peak torque was recorded from the non-dominant knee extensors using a Biodex System 4 Dynamometer (Biodex Medical Systems, Inc., Shirley, NY) in a seated position with the knee flexion angle range set at 10-100° (full extension = 0°). The participant extended the knee with maximal effort against the lever arm at a set speed of 60°/s. Six trials were completed with 30 s rest between trials, and the average of the three highest peak torque values were used for analysis.

*Maximal oxygen uptake (VO_2_max)*: VO_2_max was obtained with a ParvoMedics TrueOne 2400 metabolic measuring system with a Hans Rudolf 3813 pneumotachometer (Shawnee Mission, KS) to measure ventilation. Participants began cycling on a magnetically braked cycle Lode Corival CPET ergometer (Lode B.V., Groningen, Netherlands) at 60-80 RMPs with a starting power output of 15 watts (W) for 1 minute, and increased by 15 W every minute. VO_2_ values were collected continuously and averaged every 20 s. Participants continued cycling until exhaustion and then performed a 5-minute cool down.

*Quadriceps muscle volume*: Muscle volume was obtained via magnetic resonance imaging (MRI) scans acquired with a 0.25-Tesla musculoskeletal MRI system (Esaote G-Scan Brio, Genoa, Italy) with the isocenter positioned at mid-thigh between the patella and the inguinal crease.

*Knee extensor isometric strength*: Peak torque was quantified through three maximal voluntary contractions of the non-dominant knee extensor while seated in the Biodex with the lever arm immobilized at 90° of knee flexion. Participants had 30-60 s rest between contractions, and the trial with the highest torque value was used for analysis.

*Knee extensor isokinetic fatigue resistance*: Fatigue resistance of the non-dominant knee extensor was assessed using the same Biodex knee flexion range settings as described for *Knee extensor isokinetic strength*. Participants performed 120 consecutive knee extensions at 120°/s, and peak force of each of the contractions was summed. Summated torque from the last 40 contractions was expressed relative to summated torque from the first 40 contractions.

*Total body fat mass*: Fat mass was quantified via whole-body dual-energy X-ray absorptiometry (DXA) scans (Hologic Dixcovery QDR Model Series, Waltham, MA) using the system’s software package. Participants were scanned at the same time of day pre- and post-intervention.

Six-minute walk distance (6MW): a 6MW test was performed in a 30-meter hallway marked with cones with a left-hand turn. Distance covered in 6 minutes was used for analysis.

*Four-square step test (4SST)*: A four-foot by four-foot square was marked with athletic tape and split into quadrants. Participants stepped into each conjoining quadrant, first clockwise and then counter-clockwise. The fastest of two trials was used for analysis.

*Grip strength*: Non-dominant grip strength was assessed with a Jamar hydraulic grip strength dynamometer (Performance Health, LLC, Akron, OH). Handle position of the dynamometer was standardized for all participants at position II. Participants squeezed the handle with maximal effort for 3 s while seated with the shoulder in neutral position and the elbow flexed to 90°. Three trials were performed with 15 s rest between trials, and peak force was used for analysis.

*Five-time chair rise*: Participants started in a seated position with their arms placed across their chest. Participants then moved to standing position and returned to a seated position five times as quickly as possible.

**Exercise Intervention**

*High-intensity interval training (HIIT)*: Exercise sessions progressed from 15-30 minutes in duration throughout the study. The duration of the high-intensity intervals ranged from 15-60 seconds, and rest periods were matched at a work/rest ratio of 1:1. Exercise intensity progressively increased from 50-60% of heart rate reserve during week 1, to 85-100% of heart rate reserve during week 12.

*Moderate-intensity continuous training (MICT)*: Participants in the MICT group used the same stationary bicycle setup as in the HIIT group. Exercise sessions progressed from 20-45 minutes in duration throughout the study, while intensity progressively increased from 50-60% of heart rate reserve during week 1, to 70-75% heart rate reserve during week 12.

*Resistance training (RT)*: Participants performed 10 resistance exercises for the major muscle groups each session using free weights, machines, or body weight. Exercise intensity/duration progressively increased from 1-2 sets of 15 repetitions using a weight that elicited a rating of perceived exertion of 5-6 (0-10 scale) during week 1, to 3-5 sets of 6-20 repetitions at a rating of perceived exertion of 7-8 during week 12.

Adverse Events

Adverse events (AEs) were defined as an unexpected medical problem that happened during the course of the study related or unrelated to the intervention or assessments. All AEs occurring after informed consent was signed and until study completion were recorded. A serious adverse event (SAE) was defined as an unexpected medical problem that was believed by the investigators to be causally related to the study intervention or assessments and resulted in any of the following: a life-threatening condition, severe or permanent disability, prolonged hospitalization, or death. During the course of the study nine AEs were reported, two of which occurred during testing procedures and seven occurred during the exercise intervention. No SAEs were reported during the study. The first AE that occurred during testing was due to an equipment malfunction that resulted in prolonged, high-frequency electrical stimulation of the quadriceps muscle of one participant for approximately 4 seconds during the baseline testing Visit 3. The participant reported pain during the stimulation, but no pain 5 minutes after the incident or anytime thereafter and was able to finish all assessments, with the exception of further electrical stimulation. The second AE that occurred during testing was an abnormal heart rhythm that was detected by the exercise physiologist who was monitoring the ECG during the graded exercise test. The test was stopped, and the heart rhythm returned to normal when the heart rate approached resting levels. The participant reported no symptoms during the test or afterward, and followed up with their primary care physician following the event. This participant was removed from the study. Both of the AEs that occurred during testing were reported to the Ohio University IRB. Of the remaining seven AEs, only one was likely related to the intervention (one participant in the RT group reported mild muscle soreness for ~24 hours after an exercise session), and all were classified as *Mild*. The remaining six AEs included respiratory illness (4), knee injury occurring at work (1), and a foot injury occurring at home (1).

Adherence

All 14 participants met the minimal adherence standard of 80%, and adherence was not different between groups. The HIIT group attended 93.9% of exercise sessions (range: 86-100%), the MICT group attended 92.4% of exercise sessions (range: 89-100%), and the RT group attended 93.4% of exercise session (range: 89-97%). The one-on-one supervised training sessions ensured that target training intensities were met each session.

Stationary Bicycle Daily Output

Total output during the 12-week exercise intervention was recorded for the HIIT and MICT groups. During the first two weeks of the program both groups performed the same moderate-intensity exercise, and as such output from the first two weeks was excluded from analysis. From weeks 3-12 the HIIT group produced 94.79 ± 14.56 kJ/session, while the MICT group produced 210.83 ± 46.01 kJ/session, on average. Output from the HIIT group was 45% of the output from the MICT group.

**Supplemental Table S1. 95% Confidence Intervals for baseline and post-intervention means**

|  | HIIT  (N = 5) | |  | MICT  (N = 4) | |  | RT  (N = 5) | |
| --- | --- | --- | --- | --- | --- | --- | --- | --- |
|  | Pre | Post |  | Pre | Post |  | Pre | Post |
| Body Mass (kg) | 51.6 to 100.5 | 53.1 to 98.7 |  | 38.0 to 135.4 | 37.4 to 138.5 |  | 53.0 to 92.1 | 51.1 to 91.5 |
| BMI (kg/m^2^) | 21.5 to 35.1 | 22.1 to 34.4 |  | 22.2 to 38.5 | 22.2 to 39.4 |  | 22.4 to 32.9 | 22.1 to 32.1 |
| Isok. Strength (N-m) | 71.1 to 128.4 | 69.5 to 129.5 |  | 13.2 to 199.1 | 11.5 to 216.3 |  | 86.7 to 102.9 | 75.1 to 137.2 |
| Absolute VO_2_max (L/min) | 1.03 to 1.89 | 1.28 to 1.94 |  | 0.83 to 2.99 | 0.95 to 3.12 |  | 1.06 to 1.76 | 1.14 to 1.96 |
| Relative VO_2_max (mL/kg/min) | 17.4 to 21.4 | 17.7 to 25.7 |  | 17.3 to 27.3 | 14.7 to 33.3 |  | 16.3 to 23.1 | 17.0 to 27.3 |
| Muscle Volume (cm^3^) | 309.0 to 513.7 | 322.0 to 536.3 |  | 122.7 to 741.8 | 172.9 to 784.9 |  | 315.3 to 536.4 | 324.1 to 589.7 |
| Isom. Strength (N-m) | 73.3 to 187.1 | 82.4 to 163.3 |  | 36.6 to 223.0 | 45.1 to 208.9 |  | 89.2 to 146.5 | 108.5 to 189.0 |
| Fatigue Resistance (% of maximal) | 36.0 to 60.0 | 51.4 to 63.8 |  | 30.5 to 56.0 | 34.5 to 73.5 |  | 36.9 to 63.1 | 37.0 to 74.2 |
| Fat Mass (kg) | 18.8 to 40.4 | 19.1 to 39.7 |  | 10.2 to 52.5 | 9.5 to 52.7 |  | 16.0 to 35.8 | 15.9 to 33.5 |
| 6MW (m) | 525.6 to 610.4 | 563.8 to 658.6 |  | 498.0 to 676.5 | 504.8 to 696.2 |  | 482.3 to 632.1 | 501.5 to 668.5 |
| 4SST (s) | 5.53 to 7.29 | 5.91 to 6.94 |  | 5.09 to 7.59 | 3.77 to 7.43 |  | 4.66 to 10.02 | 4.11 to 9.09 |
| Grip Strength (kg) | 21.6 to 30.8 | 24.0 to 32.4 |  | 22.8 to 40.9 | 19.4 to 47.1 |  | 14.1 to 36.3 | 13.7 to 39.3 |
| Chair Rise (s) | 6.66 to 10.94 | 4.12 to 11.13 |  | 5.97 to 12.81 | 5.38 to 9.19 |  | 7.26 to 11.98 | 4.63 to 10.18 |

1. Tavoian D, Russ DW, Law TD, Simon JE, Chase PJ, Guseman EH, et al. A randomized clinical trial comparing three different exercise strategies for optimizing aerobic capacity and skeletal muscle performance in older adults: Protocol for the DART study. Front Med. 2019;6:236. doi:10.3389/fmed.2019.00236
